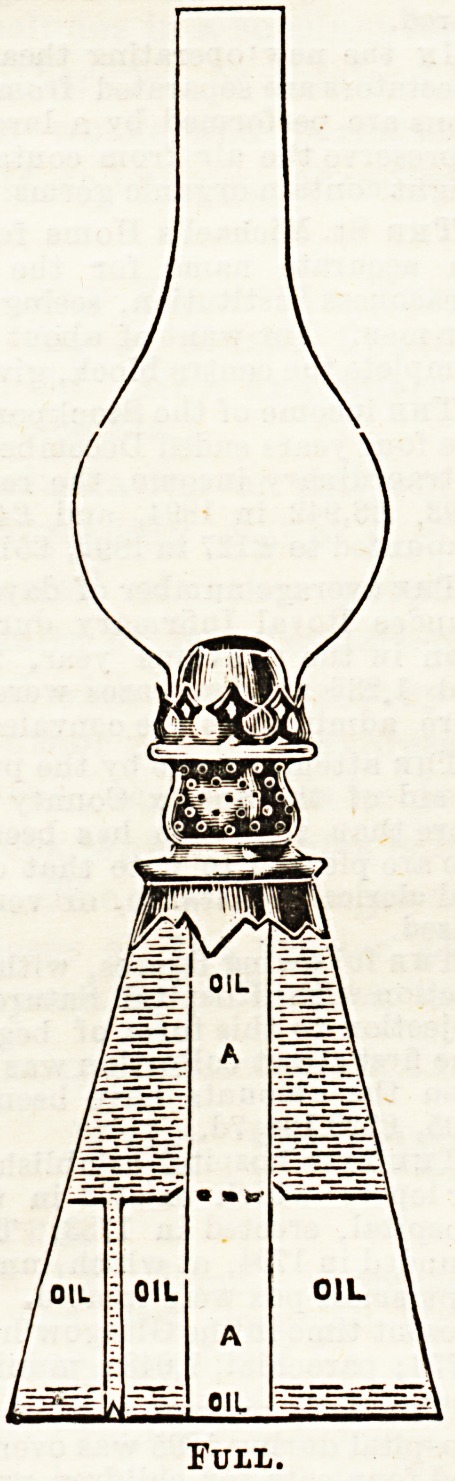# Practical Departments

**Published:** 1896-07-18

**Authors:** 


### PRACTICAL DEPARTMENTS.

### THE "HYDRO" NON-EXPLOSIVE OIL AND WATER LAMP.

The Hygienic Heating and Lighting Company, 13, Southwark Street, S.E., have brought out a lamp of a very novel character, designed to secure absolute safety from any danger of explosion. The ordinary oil lamp bursts because, as the oil is consumed, a space is left in which a highly explosive mixture of oil gas and air accumulates, which will ignite if there be any communication between it and the flame. With glass or china lamps the peril thus incurred is really very great, but accidents will also occur with metal receptacles. The principle upon which this "non-explosive, hygienic, safety, oil and water lamp" is constructed is explained in the accompanying diagrams. The wick is contained in a separate central chamber. The lamp is filled partly with water, and partly with oil, the oil supplying the

EMPTY. FULL.

oil chamber, whilst, as it is consumed, the water takes its place. There is thus no empty space left in the oil chamber, and the possibility of accumulation of oil gas is prevented. The lamp is filled in the ordinary way, and the water is said not to need changing more than "about once a year." Any kind of burner or wick may be used. So far as we have experimented with this lamp, it burns steadily and well, giving a very good light and going out immediately upon being upset. It is very inexpensive, the special lamp here illustrated being procurable for 1s. 6d., bringing it within the reach of the poorest. Taking into consideration the number of terrible accidents occurring almost daily from the bursting of common oil lamps, the inventor of this safety lamp is really a public benefactor.

## Figures and Tables

**Figure f1:**
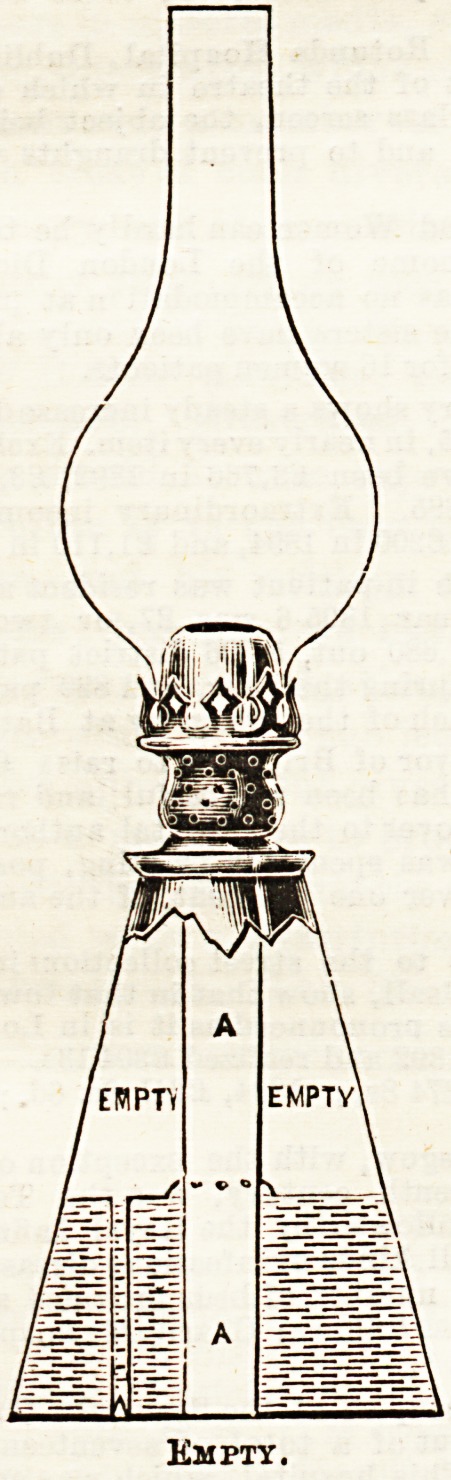


**Figure f2:**